# Late non-fasting plasma glucose predicts cardiovascular mortality independent of hemoglobin A1c

**DOI:** 10.1038/s41598-022-12034-6

**Published:** 2022-05-11

**Authors:** Yutang Wang, Yan Fang

**Affiliations:** Discipline of Life Sciences, School of Science, Psychology and Sport, Federation University Australia, University Drive, Mt Helen, Ballarat, VIC 3350 Australia

**Keywords:** Biomarkers, Cardiology, Medical research, Risk factors

## Abstract

It is unknown whether non-fasting plasma glucose (PG) is associated with cardiovascular disease (CVD) mortality. This study aimed to investigate this association in US adults. This study included adults from the National Health and Nutrition Examination Surveys from 1988 to 2014. Mortality outcomes were ascertained by linkage to the National Death Index records. Cox proportional hazards models were used to estimate hazard ratios (HRs) and 95% confidence intervals (CIs) of PG for CVD mortality. Among 34,907 participants, 1956, 5564, and 27,387 had PG from participants in early non-fasting, late non-fasting, and fasting states, respectively (defined as a period since last calorie intake of 0–2.9, 3.0–7.9, or ≥ 8.0 h, respectively). This cohort was followed up for 455,177 person-years (mean follow-up, 13.0 years), with 2,387 CVD deaths being recorded. After adjustment for all confounders including hemoglobin A1c (HbA1c), only late non-fasting PG (continuous, natural log-transformed) was positively associated with CVD mortality risks (hazard ratio, 1.73; 95% confidence interval 1.12–2.67). Higher late non-fasting PG (dichotomous, at a cut-off of 105, 110, or 115 mg/dL) was associated with higher CVD mortality risks. In addition, at the cut-off of 115 mg/dL, higher late non-fasting PG was associated with higher CVD mortality risks in those with either a normal (< 5.7%) or prediabetic HbA1c level (from 5.7 to 6.4%). In conclusion, late non-fasting PG predicts CVD mortality independent of HbA1c. Late non-fasting PG with a cut-off of 115 mg/dL may be used to identify those at high CVD risk.

## Introduction

Impaired glucose tolerance and higher fasting plasma glucose (PG) are well-known risk factors for cardiovascular disease (CVD) events^1^ and CVD mortality^2,3^. Therefore, improving glycemic control is recommended to prevent CVD in people with diabetes by the American College of Cardiology/American Heart Association guideline^4^.

Glycemic control is routinely monitored via fasting blood glucose measurement and oral glucose tolerance test^5^, both of which require patients to fast. However, fasting blood tests can be problematic for clinical practice as well as large-scale screening and epidemiological studies because they are less comfortable and less convenient for the individual compared with non-fasting tests. In addition, fasting blood tests may be less safe for people with diabetes who may experience hypoglycemia when fasting.

Hemoglobin A1c (HbA1c) is also used in the clinical management of diabetes to assess glycemic control^5^. The HbA1c assay does not need fasting and it can capture long-term high blood glucose better than fasting and 2-h oral glucose tolerance test PG^6^. However, the HbA1c assay is more expensive and less available compared to a glucose test, and this assay is unreliable in many subjects under some conditions such as smoking and various infection^5,6^.

Non-fasting PG, a simple and convenient measurement, is suggested to be a potential therapeutic target to reduce CVD mortality^7^. However, whether non-fasting PG is associated with CVD mortality is unknown. This study aimed to answer this highly clinically relevant question using US adults who attended the National Health and Nutrition Examination Survey (NHANES) from 1988 to 2014.

## Results

### General characteristics

This cohort included 34,907 adult participants, among which 1956, 5564, and 27,387 had PG data from participants in an early non-fasting (a period since last calorie intake, 0–2.9 h), late non-fasting (a period since last calorie intake, 3.0–7.9 h), and fasting state (a period since last calorie intake, ≥ 8.0 h), respectively. The mean (standard deviation, SD) age of the cohort was 46 (17) years. Other characteristics of the cohort are described in Table 1.

**Table 1:** Baseline characteristics of the cohort of 34,907 participants.

	Time since the last caloric intake (h)			Overall
	0–2.9	3.0–7.9	≥ 8.0	
Sample size	1956	5564	27,387	34,907
Age, y, mean (SD)	51 (20)	50 (19)	49 (19)	49 (19)
Sex (male), %	47.6	46.6	47.8	47.6
PG, mg/dL, mean (SD)	126 (70)	101 (38)	104 (33)	105 (37)
HbA1c, %, mean (SD)	6.0 (1.5)	5.6 (1.2)	5.6 (1.0)	5.6 (1.1)
**Ethnicity, %**				
Non-Hispanic white	39.7	45.6	44.9	44.7
Non-Hispanic black	31.5	24.4	22.1	23.0
Mexican–American	19.0	25.5	22.0	22.4
Other	9.8	4.6	11.0	9.9
**Obesity, %**				
Underweight	2.2	2.3	1.7	1.8
Normal	33.2	36.2	31.8	32.6
Overweight	33.9	35.0	34.2	34.3
Obese	28.5	26.0	31.3	30.3
Unknown	2.1	0.4	1.1	1.0
**Education, %**				
< High School	39.6	40.7	32.3	34.1
High School	26.5	29.6	25.7	26.4
> High School	33.1	29.0	41.7	39.2
Unknown	0.9	0.6	0.3	0.4
**Poverty-income ratio, %**				
< 130%	33.4	28.6	28.8	29.0
130%-349%	33.8	40.1	36.9	37.3
≥ 350%	20.7	22.8	25.9	25.1
Unknown	12.1	8.5	8.4	8.6
**Physical activity, %**				
Inactive	25.1	34.7	26.3	27.6
Insufficiently active	30.8	41.2	37.1	37.4
Active	44.1	24.1	36.6	35.0
**Alcohol consumption, %**				
0 drink/week	19.5	18.4	17.9	18.0
< 1 drink/week	13.8	11.6	22.4	20.2
1–6 drinks/week	16.1	18.1	20.1	19.6
≥ 7 drinks/week	11.9	12.0	12.8	12.6
Unknown	38.7	39.9	26.8	29.6
**Smoking status, %**				
Past smoker	29.0	24.4	22.6	23.3
Current smoker	24.5	26.2	25.1	25.2
Other	46.4	49.4	52.3	51.5
**Hypertension, %**				
Yes	36.2	29.5	31.4	31.4
No	62.8	69.8	68.0	68.0
Unknown	1.0	0.7	0.6	0.6
**Hypercholesterolemia, %**				
Yes	23.2	19.9	25.7	24.7
No	38.3	36.0	41.5	40.5
Unknown	38.5	44.1	32.7	34.9

HbA1c, hemoglobin A1c; PG, plasma glucose; SD, standard deviation.

### PG levels

**Figure 1:**
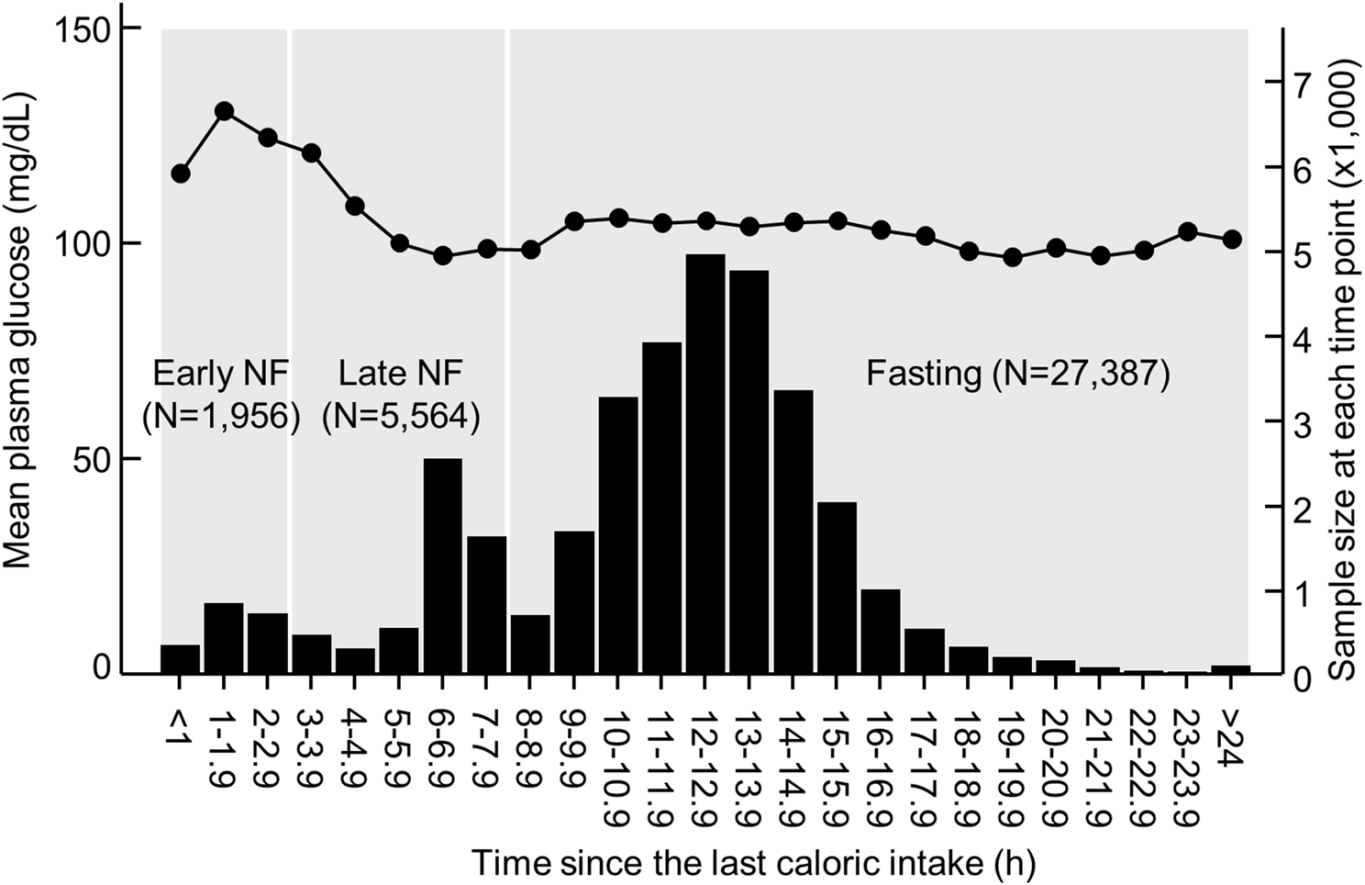
Mean plasma glucose concentrations at each hour since the last caloric intake. The line graph represents plasma glucose concentrations, and the histogram represents sample size at each time point. The time since the last caloric intake was divided into three periods: 0–2.9 h (early non-fasting), 3.0–7.9 h (late non-fasting), and ≥ 8.0 h (fasting). NF, non-fasting.

The mean (SD) PG of the cohort was 102 (31) mg/dL (Table 1). PG peaked during the second hour and then kept decreasing until the sixth hour after the last caloric intake (Fig. 1). It increased slightly during the tenth hour after the last caloric intake and then maintained relatively stable during the remaining fasting period (Fig. 1).

### Association of PG with mortality

This cohort was followed up for 455,177 person-years with a mean follow-up of 13.0 years. During the follow-up, 2387 CVD deaths and 8615 all-cause deaths were recorded.

Regardless of fasting status, PG (continuous, natural log-transformed) was positively associated with CVD mortality and all-cause mortality after adjustment for all the tested confounders except for HbA1c (Table 2). However, after further adjustment for HbA1c, only late non-fasting PG was positively associated with CVD mortality (hazard ratio [HR], 1.73; 95% confidence interval [CI], 1.12–2.67), and only late non-fasting (HR, 1.88; 95% CI 1.46–2.41) and fasting PG (HR, 1.47; 95% CI 1.23–1.76) were positively associated with all-cause mortality (Table 2). Sensitivity analyses showed that after exclusion of those who died of non-CVD causes, late non-fasting PG (continuous) remained to be positively associated with CVD mortality (HR, 2.00; 95% CI 1.28–3.10; Supplementary Table 2).

**Table 2:** Mortality risk associated with a 1-natural-log-unit increase in plasma glucose in 34,907 participants.

	Model 1		Model 2		Model 3		Model 4	
	HR (95% CI)	P value	HR (95% CI)	P value	HR (95% CI)	P value	HR (95% CI)	P value
**CVD mortality**								
Early non-fasting	3.43 (2.66–4.42)	< 0.001	1.82 (1.35–2.45)	< 0.001	1.70 (1.23–2.35)	0.001	1.09 (0.66–1.82)	0.734
Late non-fasting	4.16 (3.40–5.10)	< 0.001	2.93 (2.26–3.79)	< 0.001	2.73 (2.09–3.58)	< 0.001	1.73 (1.12–2.67)	0.013
Fasting	5.00 (4.33–5.79)	< 0.001	2.25 (1.86–2.72)	< 0.001	2.17 (1.79–2.63)	< 0.001	1.16 (0.83–1.63)	0.376
**All-cause mortality**								
Early non-fasting	3.10 (2.68–3.58)	< 0.001	1.60 (1.36–1.89)	< 0.001	1.69 (1.41–2.02)	< 0.001	1.31 (0.98–1.75)	0.070
Late non-fasting	3.69 (3.27–4.16)	< 0.001	2.35 (2.02–2.74)	< 0.001	2.26 (1.93–2.65)	< 0.001	1.88 (1.46–2.41)	< 0.001
Fasting	4.23 (3.91–4.58)	< 0.001	1.79 (1.61–1.98)	< 0.001	1.75 (1.58–1.95)	< 0.001	1.47 (1.23–1.76)	< 0.001

CI, confidence interval; CVD, cardiovascular disease; HR, hazard ratio.

Model 1: unadjusted.

Model 2: adjusted for age, sex, and ethnicity.

Model 3: adjusted for all the factors in Model 2 plus obesity, education, poverty-income ratio, survey period, physical activity, alcohol consumption, smoking status, self-reported hypertension, and self-reported hypercholesterolemia.

Model 4: adjusted for all the factors in Model 3 plus natural log-transformed hemoglobin A1c.

Sub-analyses were conducted in the sub-cohort of 5,564 participants with late non-fasting PG data. In these sub-analyses, PG was treated as a dichotomous variable (≥ vs < a cut-off value). The results showed that at a cut-off of 105, 110, or 115 mg/dL, late non-fasting PG was positively associated with CVD mortality (Supplementary Table 1). The sub-analyses also showed that higher late non-fasting PG (at a cut-off from 95 mg/dL up to at least 135 mg/dL) was associated with an increased risk of all-cause mortality (Supplementary Table 1).

Further analyses in the late non-fasting sub-group showed that, at the cut-off of 105, 110, or 115 mg/dL, higher PG (dichotomous) was associated with higher CVD mortality in those who were classified by HbA1c as normal (HbA1c: < 5.7%) or prediabetes (HbA1c: 5.7 to 6.4%, Table 3). The results remained significant for the cut-off of 115 mg/dL after exclusion of those who died of non-CVD causes (Table 4).

**Table 3:** Mortality risk associated with late non-fasting plasma glucose (dichotomous) in 5564 participants, stratified by HbA1c.

HbA1c	CVD mortality		All-cause mortality	
	HR^a^ (95% CI)	*P* value	HR^a^ (95% CI)	*P* value
**Plasma glucose ≥ 105 vs < 105 mg/dL**				
< 5.7%	2.61 (1.99–3.44)	< 0.001	1.22 (1.01–1.47)	0.037
5.7%-6.4%	1.48 (1.05–2.07)	0.023	1.37 (1.12–1.68)	0.002
≥ 6.5%	1.21 (0.71–2.04)	0.488	1.13 (0.84–1.52)	0.422
**Plasma glucose ≥ 110 vs < 110 mg/dL**				
< 5.7%	2.11 (1.51–2.94)	< 0.001	1.20 (0.95–1.53)	0.131
5.7%-6.4%	1.64 (1.08–2.50)	0.020	1.55 (1.21–1.99)	0.001
≥ 6.5%	0.95 (0.58–1.57)	0.845	1.01 (0.76–1.34)	0.945
**Plasma glucose ≥ 115 vs < 115 mg/dL**				
< 5.7%	3.50 (2.28–5.38)	< 0.001	1.58 (1.14–2.19)	0.006
5.7%-6.4%	1.82 (1.08–3.07)	0.024	1.74 (1.29–2.37)	< 0.001
≥ 6.5%	0.91 (0.56–1.47)	0.688	1.06 (0.80–1.40)	0.677

CI, confidence interval; CVD, cardiovascular disease; HbA1c, hemoglobin A1c; HR, hazard ratio.

^a^Adjusted for age, sex, ethnicity, obesity, education, poverty-income ratio, survey period, physical activity, alcohol consumption, smoking status, self-reported hypertension, self-reported hypercholesterolemia, and natural log-transformed HbA1c.

**Table 4:** Sensitivity analysis of CVD mortality risk associated with late non-fasting plasma glucose (dichotomous) in 4042 participants, ^a^ stratified by HbA1c.

HbA1c	Model 1		Model 2	
	HR (95% CI)	*P* value	HR (95% CI)	*P* value
**Plasma glucose ≥ 105 vs < 105 mg/dL**				
< 5.7%	0.91 (0.65–1.27)	0.574	0.93 (0.66–1.30)	0.651
5.7%-6.4%	1.30 (0.92–1.84)	0.141	1.22 (0.86–1.74)	0.267
≥ 6.5%	1.56 (0.96–2.55)	0.076	1.27 (0.74–2.18)	0.394
**Plasma glucose ≥ 110 vs < 110 mg/dL**				
< 5.7%	1.01 (0.65–1.57)	0.969	1.03 (0.66–1.59)	0.913
5.7%-6.4%	1.80 (1.15–2.81)	0.011	1.67 (1.06–2.63)	0.028
≥ 6.5%	1.38 (0.88–2.16)	0.161	1.06 (0.63–1.79)	0.824
**Plasma glucose ≥ 115 vs < 115 mg/dL**				
< 5.7%	2.09 (1.19–3.68)	0.011	2.17 (1.22–3.83)	0.008
5.7%-6.4%	1.94 (1.09–3.46)	0.025	1.87 (1.05–3.33)	0.034
≥ 6.5%	1.46 (0.95–2.24)	0.084	1.13 (0.68–1.89)	0.640

CI, confidence interval; CVD, cardiovascular disease; HbA1c, hemoglobin A1c; HR, hazard ratio.

^a^The late non-fasting sub-cohort included 5564 participants, among which 1522 died of non-CVD causes. This sensitivity analysis was conducted on the remaining 4042 participants after exclusion of these 1522 participants.

Model 1: adjusted for age, sex, ethnicity, obesity, education, poverty-income ratio, survey period, physical activity, alcohol consumption, smoking status, self-reported hypertension, and self-reported hypercholesterolemia.

Model 2: adjusted for all the factors in Model 1 plus natural log-transformed HbA1c.

## Discussion

Using a cohort of US adults, this study, for the first time, demonstrated that late non-fasting PG (a period since last calorie intake, 3.0–7.9 h) predicted CVD mortality independent of HbA1c. In addition, higher late non-fasting PG, at a cut-off between 105 and 115 mg/dL, predicted higher CVD mortality in those with a normal (< 5.7%) or prediabetic HbA1c level (5.7–6.4%).

This study found that PG increased slightly during the tenth hour after last calorie intake. This is consistent with a literature report^8^ that blood glucose automatically increased, likely due to gluconeogenesis, after a 10-h since last calorie intake (e.g. at 5:30 am with the last calorie intake at 7:30 pm of the previous day).

The finding of this study that late non-fasting PG predicted CVD mortality was in agreement with literature reports. A few studies have shown that PG at 1 or 2 h after breakfast^9,10^, or 2 h after lunch^11,12^ were positively associated with risks of CVD events, although CVD mortality was not investigated in those studies. Examining PG at a set time point is less practical in the clinic, large-scale screening, and large-scale epidemiological studies. The current study suggests that it is of high clinical relevance to monitor late non-fasting PG and this is also practical because the blood glucose could be conveniently measured between 3 and 8 h after a meal, rather than at a set time point or ≥ 8 h after last calorie intake.

Late non-fasting PG was more sensitive to detect people at high CVD risk than fasting PG, as the association between fasting PG and CVD mortality was no longer significant after further adjustment for HbA1c. Although some studies found that fasting PG was positively associated with CVD mortality^3,13^, others failed to find such an association^14,15^. Given that a fasting test is less comfortable and less convenient compared with a non-fasting test, monitoring late non-fasting PG may provide additional value for assessing glycaemic control.

HbA1c is positively associated with cardiovascular disease^16^ and is used for diabetes diagnosis^5^. A point-of-care HbA1c finger stick test can provide patient with HbA1c results within minutes and is well suited for areas where medical laboratories may not be accessible^17^. This test could provide results highly correlated with those from an NGSP (National Glycohemoglobin Standardization Program) reference laboratory^17^. The point-of-care HbA1c assays, which are NGSP certified and cleared by the US food and Drug Administration (FDA), are used for diabetic patients to monitor glycemic control^5^.

Late non-fasting PG was more sensitive to detect people at high CVD risk than HbA1c, as in those who were classified as normal according to HbA1c (i.e., < 5.7%), higher late non-fasting PG was associated with a higher risk of CVD mortality. The association between late non-fasting PG and CVD mortality was also significant in those who were clarified as prediabetes according to HbA1c (i.e., 5.7–6.4%). The reason that HbA1c was less sensitive compared to late non-fasting PG to detect CVD mortality is unclear. It may be due to that the HbA1c assay could be affected by many conditions such as major blood loss, smoking, and various infection; therefore, the HbA1c assay is unreliable in many subjects^6^.

The finding of this study that late non-fasting PG predicted all-cause mortality was consistent with literature reports. A few studies have shown that PG at 1 or 2 h after breakfast^9,10,18,19^, or 2 h after lunch^11^ were positively associated with risks of all-cause mortality. In addition, one study reported that non-fasting PG^20^ was positively associated with risks of all-cause mortality. The current study suggests a positive association between non-fasting PG and all-cause mortality. In addition, it revealed heterogeneity in non-fasting PG, as late non-fasting PG predicted all-cause mortality independent of HbA1c whereas the association of early non-fasting PG with all-cause mortality was HbA1c dependent.

### Strengths and limitations

A strength of this study is its large sample size (N = 34,907). Another strength of the study is its prospective study design, with the cohort being followed up for a mean of 13.0 years. This study also has a number of limitations. First, mortality outcomes were ascertained by linkage to the National Death Index records with a probabilistic match, which may lead to misclassification^21^. However, the National Center for Health Statistics employed a matching methodology offered by the National Death Index and also used other sources of mortality information to determine the best match^22^ and a prior validation study showed high accuracy (98.5%) of the matching method^23^. Second, PG was only measured at one timepoint, which may lead to misclassification. Nevertheless, in epidemiological analysis, this misclassification tends to result in an underestimate rather than an overestimate of risk due to the effect of regression dilution bias^21,24^.

In conclusion, late non-fasting PG predicted CVD mortality independent of HbA1c. Late non-fasting PG, a simple and convenient test, could be used to identify those at high CVD risk in clinic, large-scale screening, and large-scale epidemiological studies.

## Methods

### Definition of fasting status

Fasting status was defined according to the American Diabetes Association (ADA) guidelines as a period of ≥ 8 h since last calorie intake^25–28^ and non-fasting as a period of < 8 h since last calorie intake^25–28^. The non-fasting period was further divided into two sub-periods: early non-fasting (a period of < 3 h since last calorie intake)^29,30^ and late non-fasting (a period of 3–7.9 h since last calorie intake).

### Study participants

This study used data from NHANES III (1988–1994) and the subsequent eight cycles of NHANES from 1999 to 2014. These cycles of NHANES were chosen because only these NHANES cycles had mortality data available^31^. A total of 35,070 adults aged ≥ 20 years had PG data. After exclusion of those without HbA1c (N = 112), without a fasting time (N = 19), or without a follow-up time (N = 32), 34,907 participants were included in the final analysis.

### Ethical considerations

The National Center for Health Statistics Research Ethics Review Board (ERB) approved all study protocols (ERB Numbers: NHANES III, NHANES Protocol #98-12, NHANES Protocol #2005-06, and NHANES Protocol #2011-17)^21^. All procedures were performed following the guidelines of the Declaration of Helsinki. Written informed consent was obtained from all participants. The participants’ records were anonymized before being accessed by the authors.

### Measurement of plasma glucose

PG was measured using the hexokinase-mediated reaction method^32^. In brief, hexokinase catalyzed the phosphorylation of glucose to glucose-6-phosphate, and the latter compound was then oxidized to gluconate-6-phosphate by glucose-6-phosphate dehydrogenase in the presence of nicotinamide adenine dinucleotide phosphate (NADP). The rate of formation of the reduced form of NADP (NADPH) during the second reaction was directly proportional to the glucose concentration and was measured photometrically.

### Measurement of blood HbA1c

HbA1c in the whole blood was measured by an Automated Glycohemoglobin Analyzer^33^. In brief, various forms of glycohemoglobin including HbA1c were separated by high-performance liquid chromatography (HPLC), and then be detected, and quantified by an analyzer at the absorbance of 415 nm. HbA1c was calculated as a percentage of the total amount of hemoglobin present in the sample.

### Mortality from all causes and CVD

Data on mortality from all causes, heart diseases (I00-I09, I11, I13, I20-I51), and cerebrovascular diseases (I60-I69) were directly retrieved from NHANES-linked mortality files^31^. To evaluate mortality status and the cause of death, the National Center for Health Statistics conducted probabilistic matching to link the NHANES data with death certificate records from National Death Index records. The NHANES-linked mortality files used the Underlying Cause of Death 113 (UCOD_113) code to recode all deaths according to the International Classification of Diseases, 9th Revision (ICD-9) or the International Classification of Diseases, 10th Revision (ICD–10) for the underlying cause of death. Heart diseases included ischaemic heart disease (angina pectoris and myocardial infarction), heart failure, cardiac arrhythmias, cardiomyopathy, myocarditis, endocarditis, pericarditis, and valve disorders. Cerebrovascular diseases included hemorrhage stroke, ischemic stroke, occlusion and stenosis of precerebral or cerebral arteries without resulting in stroke, and other cerebrovascular diseases (e.g., cerebral aneurysm). CVD mortality included mortality from heart or cerebrovascular diseases. Follow-up time was the duration from the time when the participant was examined at the Mobile Examination Center until death, or until the end of follow-up (December 31, 2015), whichever occurred first^31^.

### Covariates

Confounding covariates included age (continuous)^34^, sex (male or female), ethnicity (non-Hispanic white, non-Hispanic black, Mexican–American, or other)^32^, obesity (underweight, normal, overweight, obese, or unknown)^35^, education (< high school, high school, > high school, or unknown), poverty-income ratio (< 130%, 130%-349%, ≥ 350%, or unknown)^36^, and survey periods (1988–1991, 1991–1994, 1999–2000, 2001–2002, 2003–2004, 2005–2006, 2007–2008, 2009–2010, 2011–2012, or 2013–2014). NHANES III was conducted in two stages, i.e., from 1988 to 1991 and then from 1991 to 1994, and the subsequent cycles of NHANES were conducted once every two years. Lifestyle confounders included physical activity (inactive, insufficiently active, or active)^21^, alcohol consumption (never, < 1 drink per week, 1–6 drinks per week, ≥ 7 drinks per week, or unknown)^37^, and smoking status (past smoker, current smoker, or other). Clinical confounders included self-reported physician diagnosis of hypertension (yes, no, or unknown), self-reported physician diagnosis of hypercholesterolemia (yes, no, or unknown)^38^, and HbA1c (continuous, natural log-transformed).

### Statistical analyses

Data were presented as mean and standard deviation for continuous variables or percentages for categorical variables. Cox proportional hazards models were used to calculate hazard ratios and 95% confidence intervals of PG for CVD mortality and all-cause mortality. PG was treated as a continuous variable (natural log-transformed) or categorical variable (dichotomous, ≥ versus < a cut-off value). Sub-analyses were conducted in subgroups stratified according to HbA1c values: < 5.7% (normal), 5.7%-6.4% (prediabetes), or ≥ 6.5% (diabetes)^5^. Sensitivity analyses of the associations between PG and CVD mortality were conducted after exclusion of those who died of non-CVD causes.

The null hypothesis was rejected for two-sided values of *P* < 0.05. All analyses were performed using SPSS version 27.0 (IBM SPSS Statistics for Windows, Armonk, NY, IBM Corporation).

## Supplementary Information

Supplementary Tables.

## Data Availability

All data in the current analysis are publicly available on the NHANES website.
