# Supplementary material for: Late non-fasting plasma glucose predicts cardiovascular mortality independent of hemoglobin A1c
Source: Sci Rep. 2022 May 11;12:7778. doi: 10.1038/s41598-022-12034-6 (PMC9095589; doi:10.1038/s41598-022-12034-6)
Supplement: Supplementary file 1 — Supplementary Tables. [file 41598_2022_12034_MOESM1_ESM.docx]

**Supplementary Material**

# Title: Non-fasting plasma glucose with a caloric restriction between three and eight hours predicts cardiovascular mortality independent of hemoglobin A1c

**Authors**: Yutang Wang, Yan Fang

**Supplementary Table 1.** Mortality risk associated with late non-fasting plasma glucose (dichotomous) in 5,564 participants

| Plasma glucose  (mg/dL) | CVD mortality | | All-cause mortality | |
| --- | --- | --- | --- | --- |
|  | HR ^a^ (95% CI) | P value | HR ^a^ (95% CI) | P value |
| ≥90 vs <90 | 0.98 (0.81-1.19) | 0.857 | 1.00 (0.91-1.11) | 0.953 |
| ≥95vs <95 | 1.12 (0.95-1.32) | 0.180 | 1.10 (1.00-1.21) | **0.040** |
| ≥100 vs <100 | 1.11 (0.93-1.33) | 0.234 | 1.15 (1.04-1.27) | **0.005** |
| ≥105 vs <105 | 1.36 (1.11-1.66) | **0.003** | 1.35 (1.21-1.52) | **<0.001** |
| ≥110 vs <110 | 1.32 (1.04-1.68) | **0.025** | 1.40 (1.22-1.6) | **<0.001** |
| ≥115 vs <115 | 1.41 (1.06-1.87) | **0.018** | 1.59 (1.35-1.87) | **<0.001** |
| ≥120 vs <120 | 1.23 (0.90-1.69) | 0.192 | 1.51 (1.27-1.81) | **<0.001** |
| ≥125 vs <125 | 1.30 (0.93-1.82) | 0.132 | 1.52 (1.26-1.85) | **<0.001** |
| ≥130 vs <130 | 1.38 (0.97-1.95) | 0.073 | 1.62 (1.33-1.98) | **<0.001** |
| ≥135 vs <135 | 1.30 (0.91-1.88) | 0.153 | 1.61 (1.30-1.99) | **<0.001** |

CI, confidence interval; CVD, cardiovascular disease; HR, hazard ratio; vs, versus.

^a^ Adjusted for age, sex, ethnicity, obesity, education, poverty-income ratio, survey period, physical activity, alcohol consumption, smoking status, self-reported hypertension, self-reported hypercholesterolemia, and natural log-transformed HbA1c.

**Supplementary Table 2.** Sensitivity analysis of CVD mortality risk associated with a 1-natural-log-unit increase in plasma glucose in 28,679 participants after exclusion of 6,228 participants who died of non-CVD causes

|  | Model 1 | | Model 2 | | Model 3 | | Model 4 | |
| --- | --- | --- | --- | --- | --- | --- | --- | --- |
|  | HR (95% CI) | *P* value | HR (95% CI) | *P* value | HR (95% CI) | *P* value | HR (95% CI) | *P* value |
| Early non-fasting | 4.51 (3.52-5.79) | <0.001 | 1.87 (1.39-2.52) | <0.001 | 1.63 (1.16-2.29) | 0.005 | 0.77 (0.44-1.38) | 0.383 |
| Late non-fasting | 5.45 (4.48-6.63) | <0.001 | 3.20 (2.46-4.17) | <0.001 | 2.99 (2.28-3.94) | <0.001 | 2.00 (1.28-3.10) | 0.002 |
| Fasting | 5.92 (5.13-6.82) | <0.001 | 2.37 (1.95-2.89) | <0.001 | 2.73 (2.25-3.30) | <0.001 | 1.52 (1.07-2.16) | 0.020 |

CI, confidence interval; CVD, cardiovascular disease; HR, hazard ratio.

Model 1: unadjusted.

Model 2: adjusted for age, sex, and ethnicity.

Model 3: adjusted for all the factors in Model 2 plus obesity, education, poverty-income ratio, survey period, physical activity, alcohol consumption, smoking status, self-reported hypertension, and self-reported hypercholesterolemia.

Model 4: adjusted for all the factors in Model 3 plus natural log-transformed hemoglobin A1c.
